# A Hospital-Based Study on the Prevalence of Keratoconus in First-Degree Relatives of Patients with Keratoconus in Central China

**DOI:** 10.1155/2022/6609531

**Published:** 2022-05-26

**Authors:** Yawen Wang, Liyan Xu, Shaopei Wang, Kaili Yang, Yuwei Gu, Qi Fan, Qing Wang, Meng Zhu, Kai Guo, Chenjiu Pang, Shengwei Ren, Dongqing Zhao

**Affiliations:** ^1^Henan University People's Hospital, Henan Provincial People's Hospital, Henan Eye Hospital, Henan Eye Institute, Zhengzhou 450003, China; ^2^Henan Provincial People's Hospital, Henan Eye Hospital, Henan Eye Institute, Zhengzhou University People's Hospital, Henan University People's Hospital, Zhengzhou 450003, China; ^3^Xinxiang Medical University, Henan Provincial People's Hospital, Henan Eye Hospital, Henan Eye Institute, Zhengzhou 450003, China; ^4^Zhengzhou University People's Hospital, Henan Provincial People's Hospital, Henan Eye Hospital, Henan Eye Institution, Zhengzhou 450003, China

## Abstract

**Purpose:**

The present study investigated the prevalence of keratoconus (KC) among first-degree relatives of KC patients in Central China.

**Methods:**

From July 2018 to March 2022, 661 first-degree relatives of 384 KC patients were included in the present study. Corneal tomography, uncorrected distance visual acuity, manifest refraction with corrected distance visual acuity, intraocular pressure, slit-lamp microscopy, and fundus examination were performed. The diagnosis of KC was based on the Belin/Ambrosio enhanced ectasia total deviation value (BAD-D value) on the Pentacam® system (Oculus GmbH). BAD-D value <1.6 was diagnosed as healthy, 1.6≤ BAD-D value <2.6 was diagnosed as suspected KC, and BAD-D value ≥2.6 was diagnosed as KC.

**Results:**

The present study included 337 (50.98%) female and 324 (49.02%) male subjects. The prevalence of KC and suspected KC in first-degree relatives was 8.77% (*n* = 58) and 29.05% (*n* = 192), respectively. The prevalence of KC was 9.70% among parents and 7.23% among siblings.

**Conclusions:**

The higher prevalence of KC among first-degree relatives of patients with KC suggests that first-degree relatives of KC are at high risk of developing KC.

## 1. Introduction

Keratoconus (KC) is a progressive disease characterized by gradual corneal thinning and ectasia, resulting in irregular astigmatism, myopia, and mild to severe impairment in the quality of vision [1,2]. The estimated global prevalence of KC is 138 per 100,000 [3]. KC typically presents in puberty and is progressive until the third to fourth decades of life [1]. Patients with KC often suffer from an enormous financial burden and a poor quality of life due to the young age of onset and severe visual impairment caused by the disease [4]. The exact pathogenesis of KC is still unknown.

Several studies have suggested links between allergy, atopy, asthma, eye rubbing, and diabetes on the one hand and KC on the other hand [5]. In addition, genetic factors play a role in the development of KC. These include its occurrence in relatives, a positive family history in 6–10% of KC cases [1], and its higher concordance rate in monozygotic twins [6]. Researchers in many countries and regions have studied the first-degree relatives of KC patients, reporting that the prevalence of KC among first-degree relatives ranges from 2.08% to 27.9% [7–23]. In China, Li et al. [7] included 48 parents of 26 patients and reported a prevalence of 2.08% among first-degree relatives in Eastern China. However, the prevalence of KC in first-degree relatives of KC patients in Central China remains unclear. In the current study, 661 first-degree relatives of 384 KC patients were included to investigate the prevalence of KC in first-degree relatives of KC patients in Central China.

## 2. Materials and Methods

This study was conducted by following the Declaration of Helsinki guidelines and approved by the Institutional Review Board of Henan Eye Hospital (ethical approval number: HNEECKY-2019 (5)). Written informed consent was obtained from each subject.

Currently, we totally invited 1,236 first-degree relatives of 389 KC patients to participate in our study, and 669 first-degree relatives underwent the following ophthalmologic examinations: bilateral corneal tomography, uncorrected distance visual acuity, manifest refraction with corrected distance visual acuity, intraocular pressure, slit-lamp microscopy, and fundus examination. Subjects with a history of other eye problems, surgery or trauma, and significant corneal scar were excluded. Subjects wearing contact lenses or rigid contact lenses were asked to stop wearing contact lenses for two weeks or rigid contact lenses for four weeks prior to examinations. Finally, 661 first-degree relatives of 384 KC patients were recruited in the current analysis, with a participation rate of 53.47%.

The diagnosis of KC was based on the Belin/Ambrosio enhanced ectasia total deviation value (BAD-D value) on the Pentacam® (Oculus GmbH). BAD-D value <1.6 was diagnosed as healthy, 1.6≤ BAD-D value <2.6 was diagnosed as suspected KC, and the BAD-D value ≥2.6 was diagnosed as KC [24].

The final prevalence was calculated in terms of the number of diagnosed subjects. According to the above diagnostic criteria, the study population was divided into three groups: the healthy group, the suspected keratoconus group (suspected KC), and the keratoconus group (KC). Descriptive statistics were used for reporting the prevalence and demographic data. Categorical variables were expressed as the number of subjects (percentage), and numerical variables were expressed as the mean ± standard deviation.

## 3. Results

Final evaluation and analysis of 661 relatives were performed, including 337 women and 324 men.  Table 1 presents the age and gender distributions of each group. The mean age of the subjects in the healthy, suspected KC, and KC groups was 35.71 ± 14.19, 41.07 ± 11.89, and 41.86 ± 11.54 years, respectively. The mean age of the participants was 37.81 ± 13.59 years, and the male-to-female ratio was about 0.96.

According to Table 2, KC was diagnosed in 58 individuals (8.77%, male: 31.03% and female: 68.97%), and suspected KC was diagnosed in 192 individuals (29.05%, male: 42.71% and female: 57.29%). The estimated prevalence of KC was 9.70% in parents, 0.00% in children, and 7.23% in siblings (Table 3). The empirical risk of KC in the present study was 9.29% in parents-offspring and 7.23% in siblings.

We summarized previous studies concerning the prevalence of KC among first-degree relatives of patients with KC and concluded that the prevalence of KC among first-degree relatives ranged from 2.08% to 27.9% (Table 4).

## 4. Discussion

A positive family history of KC is a strong indication of a genetic predisposition [25]. In the current study, the prevalence of KC in 661 first-degree relatives of 384 KC patients was 8.77%. The estimated prevalence of KC was 9.70% in parents, with 0.00% in children and 7.23% in siblings. The empirical risk of KC was 9.29% in parent-offspring and 7.23% in siblings.

The prevalence of first-degree relatives of KC in the present study was 8.77%, while previous studies have reported a prevalence of 2.08% to 27.9% in first-degree relatives [7–23]. A study on the same topic has also been reported in China. Li et al. [7] evaluated 48 parents of 26 KC patients, concluding that the prevalence of KC in these subjects was 2.08%. In the present study, the prevalence of KC in first-degree relatives was 8.77%, with 9.70% in parents, both higher than 2.08% reported by Li et al. [7]. The significant difference in the prevalence of KC in first-degree relatives in the two studies might be attributed to the sample size differences between the two studies. In studies conducted by Besharati et al. [10] (mean age: 21.0 years), Karimian et al. [13] (mean age: 32.4 years), and Awwad et al. [12] (mean age: 12.90 years), the prevalence of KC in first-degree relatives was 12.3%, 14%, and 17.5%, respectively. The prevalence of KC in these studies in first-degree relatives of KC patients was higher than that in the present study (mean age: 37.81 years). The difference in the mean age of the subjects is also one reason why the prevalence of KC in first-degree relatives in the present study is different from that in other similar studies. By summarizing the findings of previous studies concerning first-degree relatives of KC, we concluded that these similar studies used two methods to determine whether these first-degree relatives had KC or not. One was performed by self-report measures, including questionnaires, face-to-face interviews, and telephone interviews. In the other method, the relatives of patients with KC underwent a corneal topographic examination and were evaluated by ophthalmologists. Shneor et al. [17], Jordan et al. [18], Szczotka-Flynn et al. [19], Assiri et al. [20], Owens and Gamble [21], and Zadnik et al. [22] all used self-report measures to determine KC in the first-degree relatives of KC patients. The relative prevalence of KC in these studies ranged from 12.4% to 27.9%, which is significantly higher than the prevalence in studies in which relatives were examined by corneal topography and evaluated by ophthalmologists. There is a bias in the prevalence of KC obtained by self-report, affecting the study results.

The prevalence of first-degree relatives of KC patients in Israel [17], Iran [13], and Lebanon [12] was 27.9%, 14%, and 17.5%, respectively, which are higher than 2.08% reported by Li et al. [7] and 8.77% in the present study. Region and ethnic differences might have contributed to differences in the prevalence of KC in these countries and differences in the prevalence of KC in first-degree relatives of KC patients. Differences in diagnostic criteria and instruments are also important reasons why the prevalence of KC in the first-degree relatives of KC patients in this study is different from other studies. The Galilei analyzer used in the study by Gabrielle et al. [8], the Pentacam system used in this study, and the Sirius topographic device used in the study by Shneor et al. [9] are all corneal tomography techniques. In contrast, other previous studies have used corneal topography systems, including videophotokeratography, videokeratography, and Orbscan II. Corneal tomography enables earlier detection of corneal ectasia as it permits a detailed quantitative examination of both the anterior and posterior corneal surfaces. We chose the BAD-D value as the diagnostic criteria for this study because the BAD combines elevation-based and pachymetric evaluations in one comprehensive display to give the clinician a global view of the tomographic structure of the cornea [24]. The combined use of BAD, corneal tomography, and elaborate posterior corneal surface examination helps reach an early diagnosis, prepare a proper treatment plan, and achieve good therapeutic outcomes.

In this study, the prevalence of KC in first-degree relatives was 64 times higher than the global prevalence of KC. A recent meta-analysis that included >50 million individuals from 15 countries showed that the global prevalence of KC was 138/100,000 [3]. The KC prevalence in the first-degree relatives in the present study was also much higher than the 0.9% steepness prevalence in the Chinese population [26], indicating that the first-degree relatives of KC patients are at high risk of KC. The prevalence of suspected KC in this study was 29.05%, indicating that one-third of first-degree relatives exhibited abnormal corneal topography. The abnormal corneal topography might indicate that these subjects are in the subclinical phase of KC. A long-term follow-up observation should be conducted in the future. Given the higher prevalence of KC and suspected KC among first-degree relatives in this study, ophthalmologists should perform more careful preoperative screening for keratorefractive surgery candidates with a family history of KC. In addition, patients should be informed of the genetic basis of the disease, and their family members should be screened.

The prevalence of KC among parents in the present study was 9.70%, which was lower than the 14% rate reported in Gabrielle's study [8] and higher than the 2.08% rate reported by Li et al. [7]. The empirical risk of KC in the present study was 9.29% in parents-offspring, which was much higher than 2.92% in parents-offspring in a study by Wang et al. [14]. Regarding the siblings of the patients, the 7.23% prevalence in the present study and the 10% prevalence reported in Gabrielle's study [8] were both lower than the 12.3% prevalence reported by Besharati et al. [10]. The empirical risk of KC in the present study was 7.23% in siblings, which was much higher than the 3.78% rate in siblings reported by Wang et al. [14]. The prevalence of KC in the children in the present study was 0.00%, which was lower than the 3% prevalence mentioned in the study by Gabrielle et al. [8]. The discrepancy might be related to the small number of children included in the present study. In Gabrielle's study, 37% of the families included had children, while only 4.17% of the families in the present study had children because the patients visiting our hospital were younger, and most of them have no children. The prevalence of KC was not consistent across family relationship types in the present study and was also seen in the French study [8] and the study by Wang et al. [14]. Insufficient research is available on this subject, and further studies are required to provide a reasonable explanation.

In this study, only the BAD-D value provided by the Pentacam system was used as the criteria for screening KC, which might have overestimated the prevalence of KC among first-degree relatives of patients with KC. The lack of a control group was also a weakness of the study. However, the purpose of this study was not to evaluate the characteristics of corneal topography and the diagnostic efficacy of corneal topography parameters in this population. The lack of a control group did not significantly affect the results of this study. Another shortcoming of this study is that first-degree relatives of patients with KC were not fully included. Further multicenter and large-sample studies on the first-degree relatives of KC patients are necessary to validate the relevant results.

## 5. Conclusions

First-degree relatives of KC patients are at high risk of KC and should be screened for early detection of abnormal changes in corneal topography. Keratorefractive surgery should also be considered cautiously in these individuals. The study of the high prevalence of KC in first-degree relatives also provides a reference for the genetic study of KC.

## Figures and Tables

**Table 1:** General characteristics of the healthy, suspected KC, and KC groups.

	Healthy	Suspected KC	KC	Total
No. of subjects	411	192	58	661
Age (years, mean ± SD)	35.71 ± 14.19	41.07 ± 11.89	41.86 ± 11.54	37.81 ± 13.59
Male : female ratio	1.20	0.75	0.45	0.96

**Table 2:** Comparison of sexes in the healthy, suspected KC, and KC groups.

Diagnosis	Male	Female	Total	Prevalence (%)
Healthy	224 (54.50%)	187 (45.50%)	411 (100%)	62.18
Suspected KC	82 (42.71%)	110 (57.29%)	192 (100%)	29.05
KC	18 (31.03%)	40 (68.97%)	58 (100%)	8.77
Total	324 (49.02%)	337 (50.98%)	661 (100%)	

**Table 3:** Number of people diagnosed with keratoconus in different family relationship types.

Diagnosis	Parent	Sibling	Offspring	Total
Healthy	274 (57.81%)	118 (71.08%)	19 (90.48%)	411
Suspected KC	154 (32.49%)	36 (21.69%)	2 (9.52%)	192
KC	46 (9.70%)	12 (7.23%)	0	58
Total	474 (100%)	166 (100%)	21 (100%)	661 (100%)

**Table 4:** Previous studies of the prevalence of keratoconus among first-degree relatives.

Study	Country	Year	No.of relatives	Family degree	Age	Method	Prevalence (%)
Gabrielle et al. [8]	France	2020	221	First-degree	30.5	Galilei	9.05
Shneor et al. [9]	Israel	2020	56	First-degree	21.9 (6–63)	Sirius	3.6
Li et al. [7]	China	2020	48	First-degree (parents)	44.14 ± 1.70	Pentacam	2.08
Awwad et al. [12]	Lebanon	2019	177	First-degree	12.9 ± 4.5 (6–18)	Galilei	17.5
Kriszt et al. [16]	Hungary	2014	145	First-degree and others	—	TMS-4	7.6
Shneor et al. [17]	Israel	2013	—	First-degree and others	—	Self-reported	27.9
Jordan et al. [18]	New Zealand	2011	—	First-degree and others	—	Self-reported	12.4
Besharati et al. [10]	Iran	2010	150	First-degree (sibings)	21 (15–39)	Videophotokeratography	12.3
Steele et al. [15]	Australia	2008	90	First-degree and others	42.96 ± 18.50	Orbscan II	14.67
Karimian et al. [13]	Iran	2008	150	First-degree and others	32.4 ± 15 (16–83)	Videokeratography (CSO)	14
Szczotka-Flynn et al. [19]	USA	2008	—	First-degree and others	—	Self-reported	17.8
Kaya et al. [11]	Turkey	2008	72	First-degree	—	Orbscan-II	11.1
Assiri et al. [20]	Saudi Arabia	2005	—	—	—	Self-reported	16
Owens and Gamble [21]	New Zealand	2003	—	—	—	Self-reported	23.5
Wang et al. [14]	USA	2000	1226	First-degree	45.4 ± 18.6 (13–93)	Videokeratography (TMS-I)	3.3
Zadnik et al. [22]	USA	1998	—	First-degree and others		Self-reported	13.5
Ihalainen [23]	Finland	1986	—	—	—	—	19

## Data Availability

All relevant data are included in the paper and its supporting information files. Contact to Dr. Shengwei Ren (ysgzz2018@163.com) for additional information regarding data access.
